# Foaming of rhamnolipids fermentation: impact factors and fermentation strategies

**DOI:** 10.1186/s12934-021-01516-3

**Published:** 2021-03-29

**Authors:** Zhijin Gong, Ge Yang, Chengchuan Che, Jinfeng Liu, Meiru Si, Qiuhong He

**Affiliations:** School of Life Sciences, Qufu Normal University, Qufu, Shandong Province 273165 China

**Keywords:** Rhamnolipids, Foaming, Impact factors, Fermentation strategies, Large‐scale production

## Abstract

Rhamnolipids have recently attracted considerable attentions because of their excellent biosurfactant performance and potential applications in agriculture, environment, biomedicine, etc., but severe foaming causes the high cost of production, restraining their commercial production and applications. To reduce or eliminate the foaming, numerous explorations have been focused on foaming factors and fermentation strategies, but a systematic summary and discussion are still lacking. Additionally, although these studies have not broken through the bottleneck of foaming, they are conducive to understanding the foaming mechanism and developing more effective rhamnolipids production strategies. Therefore, this review focuses on the effects of fermentation components and control conditions on foaming behavior and fermentation strategies responded to the severe foaming in rhamnolipids fermentation and systematically summarizes 6 impact factors and 9 fermentation strategies. Furthermore, the potentialities of 9 fermentation strategies for large-scale production are discussed and some further strategies are suggested. We hope this review can further facilitate the understanding of foaming factors and fermentation strategies as well as conducive to developing the more effective large-scale production strategies to accelerate the commercial production process of rhamnolipids.
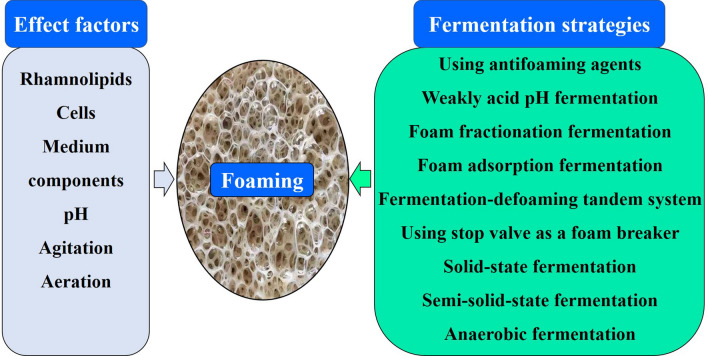

## Background

**Fig. 1:**
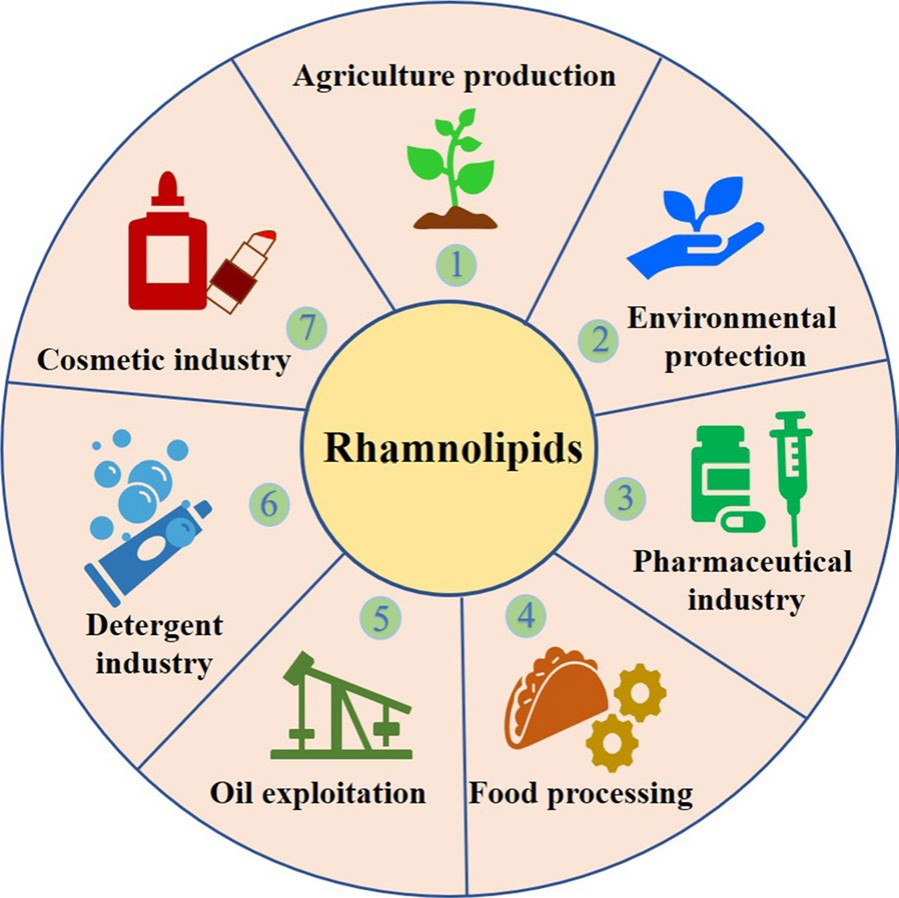
Potential applications of rhamnolipids

Surfactants including chemical synthetic surfactants and biosurfactants are a group of amphiphilic compounds that help to reduce the surface tension of a liquid or interfacial tension between two liquids [1], and are widely applied to industrial production and daily life as wetting agents, emulsifiers, foaming agents or detergents [2]. Biosurfactants are mainly produced by microbial metabolism [3–5] and are considered as potential substitutes for traditionally chemical synthetic surfactants in several industrial applications [6] because of their excellent properties, such as antimicrobial [7–9], good foaming [10, 11], emulsification [12, 13] and wettability [14], low toxicity [15, 16], biodegradation [17, 18] and produced from renewable resources [16, 19–21]. Recently, according to a market research report, the biosurfactants present a fastest-growing market and the global biosurfactants market will reach $2,889 Million by 2024, growing at a compounded annual growth rate (CAGR) of 4.4 % from 2016 to 2024 (https://www.giiresearch.com/report/var562786-biosurfactants-market-by-product-type-rhamnolipids.html). Rhamnolipids contain a hydrophilic group made up of one or two molecules of rhamnose and a hydrophobic group consisted of one or two molecules of β-hydroxyalkanoic acids (Rha–Rha–C_m_–C_n_ or Rha–C_m_–C_n_, m and n: 8, 10, 12, or 14) [22–25]. As a class of glycolipid-type biosurfactants primarily produced by *Pseudomonas aeruginosa* [26–29], rhamnolipids have a huge market demand and momentum with enormous application potential in agriculture production [30, 31], environmental protection [32, 33], pharmaceutical industry [34–36], food processing [37, 38], oil exploitation [39–42], detergent industry [43] and cosmetic industry [44] (Fig. 1). However, compared to conventional chemical synthetic surfactants, the high cost of production caused by severe foaming during fermentation limits the commercial application of rhamnolipids [15, 23].

Foam is dispersion of gas inside a fluid. The rhamnolipids biosurfactants adsorb to the air/water boundary of bubbles, reducing the surface tension of water and increasing the stability of bubbles, which permit the creation of abundantly steady aqueous foam in rhamnolipids fermentation [24]. In addition, the surface properties of cells, medium components, and fermentation conditions including pH, agitation as well as aeration also largely affects the foaming behavior of rhamnolipids fermentation [10, 15]. Therefore, the studies of the foaming factors and fermentation strategies responded to the severe foaming are crucial for solving foam problem and realizing industrial production of rhamnolipids. In fact, many researches have focused on these fields [2, 10, 15], but a systematic summary and discussion are still lacking. Herein, we focus on reviewing the foaming factors of fermentation components, control conditions as well as fermentation strategies responded to the severe foaming of rhamnolipids fermentation (Table 1). Additionally, the potentialities of these strategies for large-scale production are discussed and some further strategies are suggested.

**Table 1:** Overview of fermentation strategies involved in this review

Code	Strategies	Cultivation scale	Process	Time (h)	Production	References
1	Using antifoaming agent	5 l	Fed-batch	260	240 g/l	Bazsefidpar et al. [76]
		5 l	Fed-batch	120	70.56 g/l	Zhu et al. [16]
		50 l	Batch	100	*38.3* g/l	Sha et al. [78]
2	Fermentation in weak acid condition	2 l	Batch	217	42.1 g/l	Sodagari et al. [11]
3	Foam fractionation fermentation	2.5 l	Batch	16	0.85 g	Beuker et al. [85]
		2.5 l	Batch	30	3.99 g/l (in foam)	Willenbacher et al. [84]
		10 l	Batch	Ahout 500	70 g	Heyd et al. [80]
4	Foam adsorption fermentation	1.5 l	Batch	42	42 g/l	Zheng et al. [82]
5	Fermentation-defoaming tandem system	10 l fermenter with a 100 l foam collector	Batch	72	30 g/l	Long et al. [10]
		300 l fermenter with a 300 l foam collector	Batch	168	60 g/l	Gong et al. [23]
		2.5 l fermenter with a 0.5 l foam collector	Fed-batch	240	8.06 g/l	Salwa et al. [87]
6	Using Stop valve as a foam breaker	10 l fermenter with a 10 l foam collector	Batch	96	About 40 g/l	Long et al. [75]
7	Solidstate fermentation	30 l air pressure pulsation solid-state fermentation fermenter	Batch	168	39.8 g/l	Gong et al. [19]
		250 ml Erlenmeyer flasks	Batch	144	41.87 g/l	El-Housseiny et al. [101]
		250 ml Erlenmeyer flasks	Batch	288	45.4 g/l	Camilios-Neto et al. [61]
		250 ml Erlenmeyer flasks	Batch	288	46 g/l	Neto et al. [93]
8	Semi-solid-state fermentation	250- ml Erlenmeyer flask	Batch	288	18.7 g/l	Wu et al. [95]
9	Anaerobic fermentation	6 l	Batch	214	0.63 g/l	Zhao et al. [97]
		6 l	Batch	220	1.08 g/l	Zhao et al. [42]
		6 l	Batch	240	1.61 g/l	Zhao et al. [98]
		The extracapillary space of the hollow-fiber setup	Circulate medium in the extracapillary space	1250	About 5 g/l	Pinzon et al. [100]

## Impact factors of foaming in rhamnolipids fermentation

Gas bubbles generate from a diffusion of gas in liquid with bulk density approaching that of the gas [45]. The agglomerations of gas bubbles form foam [46]. Foam formation is a common phenomenon in the fermentation process [47–49], and has a desirable property in some fermentation production such as beer production [50, 51]. However, during the fermentation of rhamnolipids, severe foaming is not expected because it causes some adverse effects [52–54], such as reducing the working volume of fermenter [16, 23], losing the biomass and broth [23] as well as increasing the risk of contamination [2]. Therefore, in order to effectively control the foaming, it is crucial to explore the impacts of fermentation factors on foam formation.

### Rhamnolipids and cells

Although rhamnolipids have been commonly accepted as the major factor dominating the severe foaming in aerobic fermentation owing to its excellent foaming ability [10, 55, 56], the reports for systematically evaluating the contributions of rhamnolipids to severe foaming are still negligibly few. Surprisingly, in a recent study, the hydrophobic *pseudomonas aeruginosa* cells (unwashed), not (cells-free) rhamnolipids, are reported to be the primary foaming factor throughout the fermentation, even though the concentration of rhamnolipids reaches about 15 g/l [47]. Additionally, the similar result is found in a later report [57]. The severe foaming during the fermentation of rhamnolipids is attributed to the hydrophobicity of unwashed cells imparted by rhamnolipids and other metabolites adsorbed at the surface of cells. The existence of rhamnolipids and other metabolites on the cells is not considered to affect the conclusion that cells are the primary cause of broth foaming in fermentation, because from the process point of view, the integrated materials are also part of the cells [15]. However, a subsequent systematically investigation of foaming ability and foam stability of the fermentation supernatant (containing rhamnolipids) and washed cells suggested that the rhamnolipids still play a major role in the process of severe foaming in fermentation [10].

This inconsistence may be caused by the residues of rhamnolipids on the unwashed cells, which indicates that the rhamnolipids still play a major role in severe foaming, but the combination of *p. aeruginosa* cells together with rhamnolipids and other hydrophobic metabolites enhance the foaming behavior of fermentation [15]. In addition, the low concentrations of rhamnolipids solution generate large, unstable and readily collapsed bubbles, and the high concentrations of rhamnolipids generate finer and uniform bubbles with high stability and water content [10, 24]. Cells trapped in foam experience oxygen and nutrient limitations causing autolysis, which in return releases microbial proteins that enhance foaming ability [58].

### Medium components

Many aspects of the medium compositions affect foam formation, including soybean oil, fresh medium addition (except soybean oil), etc. Soybean oil as a carbon source consisted of longer chain fatty acids is widely applied in the rhamnolipids fermentation [11, 59–61], besides it has excellent defoaming properties and can compete with foaming metabolites to weaken liquid films and destabilize bubbles [62]. Thereby the foaming behavior of rhamnolipids fermentation broth is partly suppressed by the soybean oil added into medium as a carbon source. For example, the foam volume never reaches 25 % of the liquid broth volume in the soybean oil-based counterpart, but exceeds 25 % of the liquid broth volume in the glycerol-based counterpart [11]. In addition, evidences have been shown that the major function of soybean oil on defoaming was able to decrease the maximum foam volume by reducing foam stability [15]. The foaming properties of two broth samples with or without fresh soybean oil present similar initial foaming rates but the maximum foam volumes are remarkable dissimilarity: 47 ml for the broth with fresh soybean oil and 24 ml for the broth without fresh soybean oil.

The fresh medium addition can change the surface properties of cells (e.g., increased hydrophobicity) as a result of rapid adsorption of some fresh medium components on the cells surface [15] or lead to the protein solubility decreases with increasing salt concentrations caused by supplementing fresh medium and raise adsorbed protein concentrations in the foam layer, which in turn increases foaming ability [58]. The fresh medium addition causing immediate increase in froth foaming is repeatedly observed in rhamnolipids fermentation [15]. In addition, the use of a Ca-free medium and the addition of the trace elements solution may inhibit the cells growth, so as to avoid higher foam formation associated with cells growth at earlier fermentations [6, 11, 63].

### pH

Bacterial cells are charged with some charged biomolecules appearing at their cell walls, like lipoproteins, peptidoglycan, etc. [15, 64], besides the reported pKa of rhamnolipids is approximately 4.3 to 5.5 [65, 66]. Therefore, the electrostatic repulsion between charged cells or between rhamnolipids molecules adsorbed inner and outer membranes of bubbles are weaken owing to the decrease of pH, which causes the reduced net negative charge on film surfaces and accelerates the coalescence of bubbles as well as leads to slower and more unstable foaming [67]. On the other hand, the aggregate morphology of rhamnolipids can be reversibly altered from vesicles to lamella, lipid particles, and finally to micelles under weakly acidic conditions within a narrow pH range of about 5–7, affecting the foaming ability of rhamnolipids [66]. Evidences have been shown that the foaming ability of rhamnolipids fermentation decreases by approximately 80 % when pH is lowered from 6.7 to 5.0 [11]. The foaming rate of the purified rhamnolipids solutions is significantly reduced when pH is lowered from pH 9 to pH 3 [24, 68]. In addition, heat causes nitrogen sources to become hydrolyzed, leading to Maillard reactions between reducing sugars and amino acids or proteins. Maillard reaction products enhance foam formation, especially at higher sterilization pH values. For example, during sterilization, the decrease of the pH value from 5.2 to 4.0 and 3.0 reduces foaminess of the medium (3 % glucose + 5 % potato protein liquor) from 737 to 66 and 45 s [69].

### Agitation

Agitation often increases foam by increasing air entrapment and cells lysis. As stirring speed increased, foam cells size decreases and becomes more stable, which in turn increases the rate of foam buildup [58]. For example, compared with the foam generated under no stirring, the foam stability and water content increase from 20 min and 1.83 % to 60 min and 5 % under the stirring speed of 300 rpm in a 10 l bioreactor contained rhamnolipids solution. In addition, according to the morphologic observation, the large and transparent bubbles are produced under no stirring while the much finer and uniform bubbles are obtained at stirring speed of 300 rpm [10]. The bubbles produced via intense stirring have higher stability and possess the feature of wet films and a small and narrowly distributed bubble diameter and are more difficult for foam control, which may be due to that the intense stirring provides a high shear force, engendering fine bubbles and breaking the large bubbles into small and stable secondary foam according to the theory of secondary foam formation. In addition, compared with polysorbate (Tween 20), a weak foaming agent, rhamnolipids exhibit low foam stability under no stirring but can significantly aggravate foaming issues under stirring [10, 70, 71]. Hence, reducing stirring speed will be an appropriate approach to weaken foaming behavior and thus facilitate foam control. Futhermore, the extensively used mechanical foam breaker which is fixed in the headspace of the bioreactor performs well in the foam control of convention submerged fermentation but it should be avoided in rhamnolipids fermentation, because mechanical high-speed foam breaker aggravates the secondary foam, forming a dense air emulsion layer. Evidences have been shown that, after removing the foam breaker, none of these dense secondary foams is observed [2, 10].

### Aeration

In the context of fermentation, the fermentation reactor can be conveniently divided into two zones, liquid zone and foam phase, in which the dispersed air properties are very different. The core of the fermentation reactor is the liquid zone where the fermentation processes takes place and in which dispersed air is presented as air bubbles, providing a source of oxygen. Large amount of rising bubbles cause collisions between bubbles. Bubbles coalescence result in fewer, larger bubbles, and then the growing bubbles break in the liquid zone. If the rate of bubbles rupture at the liquid zone is slower than the rate of air injection into a fermentation reactor, the volume of foam phase gradually increases with time [72]. Therefore, high aeration flux, coupled with foam-stabilizing products present in the broth, such as proteins and carbohydrates, makes fermentation processes prone to foaming. In the fermentation of rhamnolipids, rhamnolipids have more excellent foaming ability than proteins and carbohydrates, thus high aeration flux can more significantly enhance the foaming behavior of rhamnolipids fermentation. Evidences have been shown that, the average foaming rate of rhamnolipids solutions is approximately 3.5 ml/s at the aeration rate of 0.2 l/min compared with 1.5 ml/s at the aeration rate of 0.1 l/min [24]. The foam volume never reaches 25 % of the liquid broth volume at the 5 % DO (low aeration flux), but is more than 50 % of the liquid broth volume at the 30 % DO (high aeration flux) [11]. In addition, during our previous study, for avoiding foam escape from 300 l fermentation tank, the aeration flux of fermentation of rhamnolipids must be reduced from 150 l/min to 5 l/min during the later stages of fermentation [23].

## Fermentation strategies responded to the severe foaming

Rhamnolipids are kinds of biosurfactants with excellent foaming properties, so the foaming of fermentation is much difficult to control than that of other products [73], which brings a huge challenge for rhamnolipids production [74]. To solve this problem, different fermentation strategies have been developed [75]. Although these strategies cannot completely solve this problem, they provide a basis for further realizing efficient large-scale production of rhamnolipids.

### Using antifoaming agents

Antifoaming agents can destabilize the liquid film by various mechanisms. For example, antifoaming agents can displace the adsorbed surfactants on the film surface or rapidly spread onto the surface of the film, leading to the liquid to be squeezed away and the film to be thinned as well as causing liquid film to collapse [2]. Hence, antifoaming agents like silicon oil are commonly employed to eliminate foaming in fermentation [16, 76, 77], but they are usual insufficient to suppress the severe foaming generated from rhamnolipids fermentation [78]. In addition, the use of a great quantity of chemical antifoam agents are harmful to cells growth and rhamnolipids productivity, meanwhile adding to the complexity and costs of the downstream processes [2, 78]. Therefore, normally, antifoam agents are not single-handed employed to suppress the foaming in rhamnolipids fermentation, but are combined with other defoaming strategies like fermentation-defoaming tandem system [23]. Even if there are some reports that only antifoaming agent is used for controlling the foaming of rhamnolipids fermentation, the effective working volume of bioreactor is normally less than 50 %. For example, Chen et al. carried out the fermentation of rhamnolipids in a 5 l fermenter with a final 2 l of fed-batch fermentation volume [77]; Zhu et al. inoculated 2.5 l of initial fermentation medium into a 5 l fermenter [16]; Bazsefidpar and co-workers used a 5 l fermenter with working volume of 2 l [76]. Furthermore, in addition to commercial antifoam agents like silicon oil aforementioned, the ethanol with the least toxicity and as a carbon source of *P. aeruginosa*, is elucidated to be a promising antifoam agent used in rhamnolipids fermentation [78].

### Weakly acid pH fermentation

The weakly acid pH can remarkably affect the foaming behavior of rhamnolipids fermentation through altering electrostatic repulsion and aggregation behaviors of rhamnolipids molecules. Therefore, the attempts to suppress the foaming behavior of fermentation broth by controlling pH at 5.5, 5.7, 6.0 and 6.7 are implemented and the results suggested that the foaming is suppressed and the maximum cells concentration and the average specific productivity of rhamnolipids have also no significant difference. Thus, in order to maximize the cells growth and rhamnolipids productivity and minimize the effects of foaming, the fermentation is recruited at pH 5.5–5.7 [11]. Nevertheless, for most of rhamnolipids fermentation, the strategy of controlling pH under weakly acid conditions are unsatisfactory, because although the foaming ability of rhamnolipids fermentation broth is conspicuously eliminated, the cells growth and rhamnolipids productivity are also remarkably inhibited [2, 15]. The reduced cells growth and rhamnolipids productivity can be attributed to the abundantly dissociated hydrogen ions in broths are readily entered into cells cytoplasm, increasing the intracellular acidity and leading to DNA damage and denaturation of essential enzymes, which causes impaired cells growth and reduced rhamnolipids synthesis [79]. Additionally, the extracellular particles containing medium components, such as waxy particles, are readily formed under weakly acid conditions [68], impeding absorption of nutrient substances and rhamnolipids synthesis. Therefore, a more efficient approach for production of rhamnolipids under weakly acid conditions remains to be developed.

### Foam fractionation fermentation

Traditional mechanical defoaming techniques like using rotary devices cannot effectively suppress the severe foaming in rhamnolipids fermentation [2], thus some unconventional fermentation techniques are developed. Foam fractionation is one of the emerging technologies for target product recovery and enrichment from foam with several outstanding features such as low cost and in situ product concentrating and recovery [80–82]. In the process of foam formation, rhamnolipid molecules preferentially are adsorbed at the membrane of bubbles when the water in the films surface of bubbles is drained by gravitational force, resulting in a higher concentration of the rhamnolipids in the films surface [83]. Previous study suggested that the concentration of biosurfactants in the collapsed foam is approximately 50 times higher than that in culture medium [81]. Therefore, the foam fractionation technique can provide a high biosurfactant recovery efficiency and a high enrichment ratio. Meanwhile, using foam fractionation technique never minds the foaming problem, because foaming is conducive to recovering the target product. For these advantages, the foam separation technique is considered a promising method for solving the severe foaming in rhamnolipids fermentation. According to the previous study, a simple integrated foam fractionation process is established, that is, the foam is channeled through the exhaust cooler into traps collecting bags of foam for recovery of rhamnolipids. Through characterizing the recovery, specific and volumetric productivities of foam fractionation process, the high efficiency of the foam fractionation for rhamnolipids fermentation is elucidated [84, 85].

Although foam separation with collecting bags takes full advantage of foaming properties of rhamnolipids and avoids using antifoaming agents, there are still some insufficiencies. The primary one is the loss of biomass during fermentation. A simple integrated foam fractionation process can be conducted with the low concentration of biomass in foam, otherwise, the loss of cells has an essential impact on production efficiency [57]. Therefore, there is necessity for some kinds of strategies to prevent cells from being entrapped by the foam. A foam fractionation method with preventing loss of the cells through immobilizing *P. aeruginosa* cells in magnetic alginate beads is developed [80]. In this system, the magnetic alginate beads containing cells are retained from the foam through high gradient magnetic separation and back-flushed in the fermenter at constant intervals. After four production cycles, 70 g of final rhamnolipids amount is yielded with an average enrichment ratio of 15 in the collapsed foam, which unravels the feasibility of a continuous production of rhamnolipids by foam fractionation coupled with magnetic immobilizing cells.

**Fig. 2:**
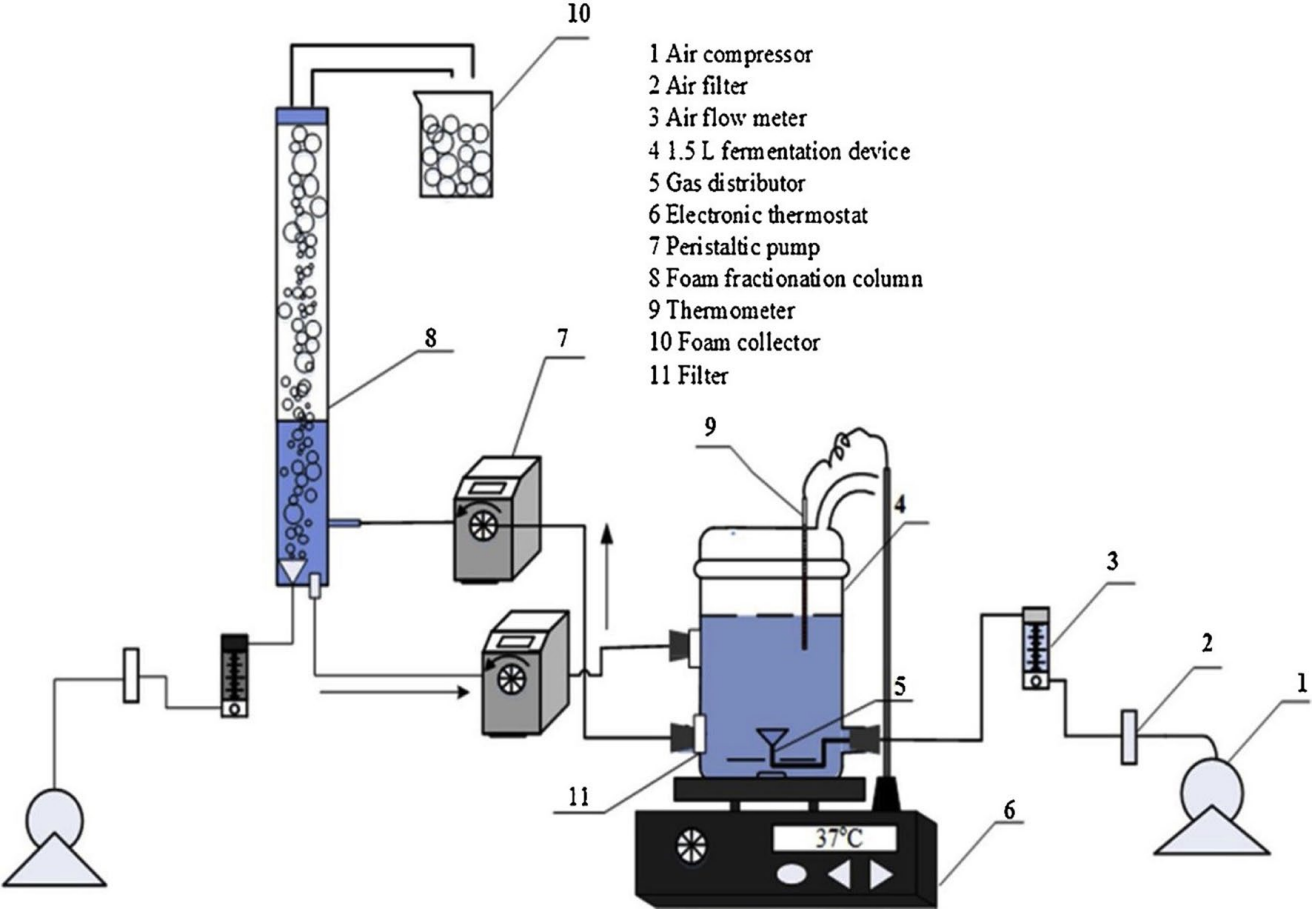
Fermentation coupling foam fractionation device for production of rhamnolipids [82]

Additionally, a novel device of cyclic fermentation coupled with foam fractionation to continuously produce rhamnolipids is developed recently [82]. The fermentation is conducted in 1.5 l fermenter at the initial stage of fermentation. When the set time is reached, the fermentation broth is pumped into the foam fractionation column, the cells are filtered and stored in the fermenter by membrane, after the loading liquid volume is reached, the sterile air is introduced into the foam fractionation column to generate foam for recovering rhamnolipids. The experimental setup is presented in Fig. 2. Through this device, the cyclic fermentation and foam fractionation are achieved without cells in the foam. Simultaneously, the start foam separation time is accurately controlled for facilitating the production of rhamnolipids and improving the productivity of fermentation.

### Foam adsorption fermentation

In order to solve the foam problem of rhamnolipids fermentation, recently, the integrated foam adsorption fermentation that can adsorb the rhamnolipids in foam and recycle cells-containing foam collapse fluid is developed [57, 86]. In this system, an automated adsorption unit is connected to between the outlet of exhaust-gas line and bottom inlet of foam container. The automated adsorption unit is packed with the hydrophobic C18 silica-based adsorbent ODS-A with enough large spherical particles to ensure enough space for cells and nutrient broth to flow through the fixed bed of the adsorbent, meanwhile capturing the rhamnolipids flowing through the surface of the hydrophobic adsorbent. The collapsed foam liquid containing cells and broth is recycled by peristaltic pump. The rhamnolipids adsorbed on the adsorbent can be eluted, and then the column wash for column recirculation is performed [57]. Through the integrated foam adsorption system, production and capture of rhamnolipids are simultaneously performed during fermentation, reducing the feedback inhibition of product and enhancing rhamnolipids enrichment (purity) and recovery efficiency for the downstream processing. Nevertheless, considering the high cost of hydrophobic C18 silica-based adsorbent, a cheap alternative may still need to be developed. Furthermore, when high cells density cultivations are established, high rhamnolipids concentrations in the foam may exceed the column adsorption capacity. In this case it will be necessary to optimize the operating conditions such as column size, number and adsorption time to ensure the sufficient adsorption capacity for rhamnolipids recovery.

### Fermentation‐defoaming tandem system

Fermentation-defoaming tandem system refers to two tanks, one is a regular fermenter that can normally detect related fermentation parameters, like DO, pH, temperature, etc. and the other is general a foam collector without the function of parameters detection [10, 23, 87, 88]. The two fermentation tanks are linked by pump, pipes and valves. The overflow liquid and escaped foam from the regular fermenter will be gathered in foam collector, and defoaming is achieved at foam collector by adjusting pressure and stirring, and then the foam collapse fluid is pumped back to the regular fermenter for fermentation. According to our previous study, the fermentation-defoaming tandem system is proven to be a promising strategy for solving the problem of foaming and efficient production of rhamnolipids. For example, in our previous study, a fermentation-defoaming tandem system with a 300 l regular fermenter and a 300 l storage tank is employed for rhamnolipids fermentation scale-up [23], and 60 g/l of rhamnolipids is obtained with the yield of 80 %. In addition, the fermentation-defoaming tandem system with a 10 l fermenter and a 100 l foam collector is used to carry out rhamnolipids fermentation and the rhamnolipids production reaches about 30 g/l at 72 h [10].

### Using stop valve as a foam breaker

Rhamnolipids foam can be readily disrupted while crossing a tiny opening of ball valve. Based on the phenomenon, an ex-situ defoaming system with stop valve to solve this problem is developed in rhamnolipids fermentation. This system is similar to the fermentation-defoaming tandem system abovementioned [10], but the difference is that the stirring impeller for mechanical defoaming is replaced by a stop valve with a diameter of 20 mm as foam breaker. The inlet of stop valve is connected to the exhaust-gas line of the fermenter and the outlet of stop valve is installed on the top of the foam collector, a pressure gauge is connected to the upper part of stop valve to reflect the opening of stop valve. When flowing through the stop valve, the foam is disrupted by a high shear rate in combination with fast separation of air from the broken foam and the foam collapse fluid with only little foam is transferred back to the fermenter for the fermentation once again. Using the Stop valve as a foam breaker, more than 90 % of the foam is disrupted and the productivity of rhamnolipids enhances 83 % compared with the fermentation-defoaming tandem system [75]. The reason for increasing productivity may be that the rapidly collapsing (within a few seconds) foam can be pumped back to the fermenter quickly, which is conducive to reducing detention time and decreasing the effects of limited mass and oxygen transfer on the cells growth in foam collector [75].

### Solidstate fermentation and semi‐solid‐state fermentation

Solidstate fermentation (SSF), non-emerging free-flowing water, is a fermentation method different from liquid-state fermentation (LSF) [89]. SSF has gained much interest in recent years because of several advantages over LSF, such as less requirements for water, energy and aeration [90–92]. More importantly, even in a forcefully aerated fermentation process, SSF will not produce foam. Hence, for solving the foaming problem, SSF is employed to produce rhamnolipids. In the SSF of rhamnolipids, the agro-industrial by-products, such as wheat straw, rice straw and sugarcane bagasse, are generally employed as the supports, and the yield of rhamnolipids is usually more than 40 g/l [61, 93]. However, the supports of agro-industrial by-products employed bring a large number of impurities into fermentation medium, increasing the difficulty for down-stream purification processing. Furthermore, these agro-industrial by-products used tends to form agglomerations, resulting in a poor transfer of mass and heat and hindering the heat transfer as well as reducing the transfer rate of oxygen and nutrients in large-scale production [19]. For solving these problems, in our recent study, a novel SSF process that the air pressure pulsation solid-state fermentation (APP-SSF) with using high‑density polyurethane foam, an artificial inert porous material with low impurities, high mechanical strength and recycled property, as SSF supports was developed for rhamnolipids production by *P. aeruginosa* [19]. The results indicated that the novel SSF process has a high productivity, less impurities and more efficient fermentation scale-up (30 l), and is a satisfactory alternative to the traditional SSF of using the agro-industrial by-products as supports.

Semi-solid-state fermentation (SSSF) is a special SSF in which the free-flowing water is contained for facilitating nutrient availability and fermentation control [94]. *P. aeruginosa*, the commonly production strain of rhamnolipids, prefers a higher water activity environment. Hence, in term of water activity, SSSF is more suitable for the production of rhamnolipids compared with SSF. Additionally, the SSSF can effectively reduce the foaming behavior in rhamnolipids fermentation. In a recent study, SSSF is developed to produce rhamnolipids used the rapeseed meal and wheat bran as matrix, and the rhamnolipids yield reaches 18.7 g/l [95]. The rhamnolipids obtained from SSSF have a satisfactory performance for restoring the heavy metal contaminated soil. Furthermore, the SSSF does not need sterilization and is readily carried out in rough conditions. These indicated that the SSSF has the potential for directly using waste products to produce rhamnolipids for inhibiting the crop pathogens and restoring soil in the countryside.

### Anaerobic fermentation

*P*. *aeruginosa* is a kind of facultative bacteria growing in aerobic or anaerobic environment and can produce rhamnolipids in anaerobic fermentation without foaming [74, 96, 97]. However, the production of anaerobic rhamnolipids fermentation is typically low [98]. For example, the strain *P. aeruginosa* SG is employed to produce rhamnolipids by anaerobic fermentation and the production of rhamnolipids (0.68 g/l) is significant less than the aerobic production of 11.65 g/l. This may be due to the expression down-regulated of several required genes for the synthesis of rhamnolipids, such as *rhlAB* and *rhlC* [96]. In addition, in order to avoid respiratory limitation under anaerobic fermentation, the denitrification is utilized as a respiration route to produce rhamnolipids, but the specific productivity is merely approximately one-third that of the aerobic fermentation [99].

The free-cells aerobic fermentation is still challenging in large-scale production of rhamnolipids, because severe foaming affects the productivity of rhamnolipids. Immobilized systems in aerobic fermentation are difficult for continuous rhamnolipids production due to oxygen transfer limitation. A continuous rhamnolipids production system combining immobilized cells and anaerobic denitrification of *P. aeruginosa* is established for avoiding the severe foaming and oxygen transfer limitation [100]. In the system, the polysulfone of 0.1 mm pore size is sealed within the cartridge case as a hollow-fiber bioreactor. The medium flows through the inside of the fibers and then is pumped back to original flask. The outside surface of fiber in the extracapillary space provides a place for cells growth. The pH, NaNO_3_ and glycerol are controlled for maintaining normal continuous fermentation. The coupled system using denitrification-based immobilized approach completely avoids the oxygen transfer limitation and foam problem, and the specific productivity of continuous rhamnolipids production reaches 0.017 g/ (g dry cells)-h.

## Perspectives for large‐scale fermentation of rhamnolipids

Conventional chemical defoaming methods like using silicon oil have been proved to be ineffective to suppress the severe foaming in rhamnolipids fermentation, unless sacrificing the working volume of fermenter and excessively using chemical antifoam agents. However, sacrificing working volume and excessively using chemical antifoam agents are not expected in large-scale fermentation because they are sharply increased the costs of fermentation and purification [78]. Therefore, the most effective strategy for chemical antifoam agent is used as an auxiliary defoaming reagent in other defoaming strategies.

In terms of rhamnolipids fermentation of *P. aeruginosa*, generally, the foaming behavior, cells growth and rhamnolipids synthesis can be remarkably suppressed at pH 5.5-6.0 [11, 15], but the strain of *P. aeruginosa* E03-40 shows no significant distinction in maximum cells concentration and average specific productivity [11], which may be due to the tolerance different of production strains to acid environments. Furthermore, considering the fact that pH 5.5-6.0 is relatively mild environments for bacteria growth. Consequently, through strain screening or metabolic engineering methods like global transcription machinery engineering to enhance the tolerance and productivity of rhamnolipids production strains at pH 5.5-6.0 will be promising strategies to solve the sever foaming problem in large-scale production.

Foam separation technologies result in the massive loss of production strains and nutritional components, and their influence on large-scale industrial production cannot be ignored [57]. Although the magnetic immobilizing cells [80] or filter [82] can avoid the cells loss in foam fractionation fermentation, they have to solve the problem of massive preparing immobilizing cells or avoid the filter clogging in large-scale production. Foam adsorption fermentation used the hydrophobic C18 silica-based adsorbent ODS-A as adsorbent is a novel separation method and can be efficiently applied for simultaneous production and recovery of rhamnolipids with a constant system productivity by recirculating cells and culture broth [57]. Additionally, the high efficiency adsorption capacity and simple purification method reduce the cost of rhamnolipids production. In future, through developing a cheaper alternative for hydrophobic C18 silica-based adsorbents and optimizing the adsorption columns size, number and adsorption time to ensure the sufficient adsorption capacity for recovery of rhamnolipids, the large-scale production of rhamnolipids used the foam adsorption fermentation may be realized.

To the best of our knowledge, the largest scale of rhamnolipids fermentation reported is the fermentation-defoaming tandem system used a 300 l regular fermenter and a 300 l storage tank [23]. The fermentation-defoaming tandem system is simple and only need to extra connect a storage tank and a circulating pump on the regular fermenter, and the production and the yield reach 60 g/l and 80 %, respectively. In addition, based on the consultation with local fermentation factory, the increased costs of defoaming in a 30 m^3^ fermentation-defoaming tandem system including equipment, wage, water, electricity, steam, machine repair and others are about 5 %-10 % of the total cost of production. Therefore, considering the yield, cost and operability, the fermentation-defoaming tandem system may be the most probable strategy to realize large-scale production of rhamnolipids at present.

Using stop valve as a foam breaker can fast disrupt foam and reduce detention time of cells in foam collector, enhancing productivity of rhamnolipids fermentation. However, the stop valve may be ineffective for defoaming, when the fermenter scale exceeds 1 m^3^ [75]. Because massive rhamnolipids foam generated needs to widen slit in the stop valve, which causes a remarkable decline of foam breaking ability. Additionally, it is impractical to use a large number of small valves in industrial fermentation process. The decline of foam breaking ability in large-scale of fermentation attributes to the widened slit reducing the pressure of slit outlet, and thus reducing shear rate and separation ability of air from the broken foam. Therefore, increasing the pressure of foam outlet to improve the defoaming efficiency is worth of being attempt in further research.

SSF can completely avoid foaming and has high production in laboratory studies [61, 93]. However, the agro-industrial by-products used restrict the large-scale application of SSF. Although, in our previous study, using the APP-SSF with high‑density polyurethane foam as an inert support can effectively improve heat and mass transfer in a 30 l fermenter [19], the application in large-scale fermentation is still unclear and needs to be remedied in further research. Rhamnolipids from SSSF with rapeseed meal and wheat bran as matrix have a promising potential for rough application like inhibiting pathogens and restoring heavy metal contaminated soil [95]. However, for large-scale extraction of rhamnolipids, using rapeseed meal and wheat bran as matrixes are adverse, because a large number of impurities are introduced, increasing the difficulty of rhamnolipids purification. The artificial inert porous polyurethane foam may be a satisfactory substitute for avoiding impurities in SSSF and the further study remains to be elucidated.

Up to now, the industrial preparation of rhamnolipids by anaerobic fermentation is usually not feasible because of the remarkable low productivity [96, 98, 99]. However, the anaerobic fermentation of rhamnolipids performs well in oil recovery through in-situ culture of rhamnolipids production strain in oil field [97, 98]. In addition, for improving the rhamnolipids production capacity in anaerobic fermentation, the genetically engineered production strain should be constructed by metabolic engineering and synthetic biology strategies.

## Conclusions

The studies of the foaming factors and fermentation strategies responded to the severe foaming in rhamnolipids fermentation are essential for solving foaming problem and realizing large-scale industrial production of rhamnolipids. For the foaming factors, fermentation components and control conditions can significantly affect the foaming behavior of rhamnolipids fermentation. Among these, the combined action of cells, rhamnolipids and other hydrophobic components may be the main contribution for the severe foaming and the further study of mechanism remains to be elucidated. For the fermentation strategies, 9 fermentation strategies are summarized and discussed in this review. Among these, the fermentation-defoaming tandem system may be the most possible to realize industrial production of rhamnolipids at present. Additionally, integrating the available advantages of different fermentation strategies to develop a novel and high efficiency fermentation-defoaming coupling system should be considered in the further study.

## Data Availability

Not applicable.

## Abbreviation

CAGR: Compounded annual growth rate
DO: Dissolved oxygen
SSF: Solidstate fermentation
LSF: Liquid-state fermentation
SSSF: Semi-solid-state fermentation
APP-SSF: Air pressure pulsation solid-state fermentation
